# Supplementary figures and images for: The dual-specificity phosphatase JSP1 regulates neutrophil adhesion via integrin-SRC signaling in vascular inflammation
Source: J Biol Chem. 2026 Mar 16;302(5):111369. doi: 10.1016/j.jbc.2026.111369 (PMC13090511; doi:10.1016/j.jbc.2026.111369)

A

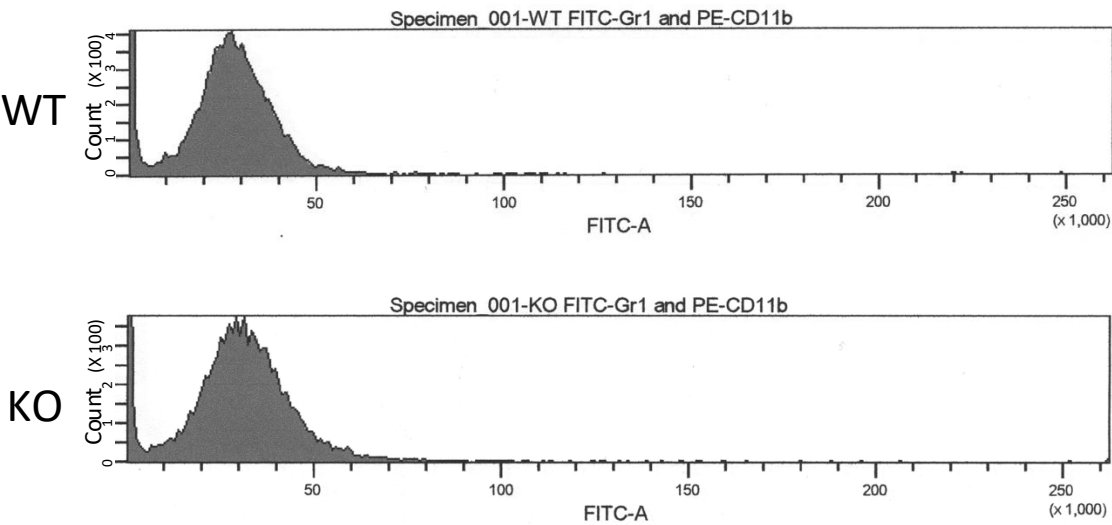

B

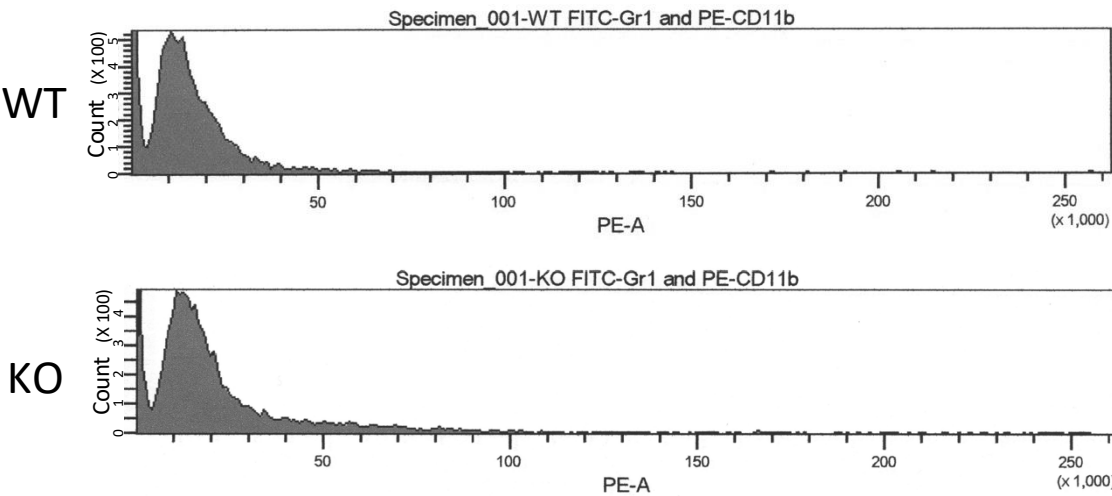

Supplementary Figure 1.

Supplement: Figure S1 [file mmc1.pdf]
