# Supplementary material for: The dual-specificity phosphatase JSP1 regulates neutrophil adhesion via integrin-SRC signaling in vascular inflammation
Source: J Biol Chem. 2026 Mar 16;302(5):111369. doi: 10.1016/j.jbc.2026.111369 (PMC13090511; doi:10.1016/j.jbc.2026.111369)
Supplement: Figure S1 legend [file mmc2.pdf]

Supplementary Figure 1. The absence of JSP1 did not alter the quantity of mature neutrophils or the expression of integrin subunit CD11b. Bone-marrow derived neutrophils from either WT or JSP1-knockout mice were double immunostained for Gr-1, a marker of mature neutrophils, and integrin subunit CD11b. (A) Flow cytometry analysis to illustrate the surface expression of Gr-1, indicating the quantity of the mature neutrophils. (B) Flow cytometry analysis to illustrate the surface expression of CD11b, indicating the expression levels of the integrin subunit. Flow cytometry histograms show fluorescence intensity (x-axis) versus cell count (y-axis).
